# A Genome-Wide Screen for Dendritically Localized RNAs Identifies Genes Required for Dendrite Morphogenesis

**DOI:** 10.1534/g3.116.030353

**Published:** 2016-06-01

**Authors:** Mala Misra, Hendia Edmund, Darragh Ennis, Marissa A. Schlueter, Jessica E. Marot, Janet Tambasco, Ida Barlow, Sara Sigurbjornsdottir, Renjith Mathew, Ana Maria Vallés, Waldemar Wojciech, Siegfried Roth, Ilan Davis, Maria Leptin, Elizabeth R. Gavis

**Affiliations:** * Department of Molecular Biology, Princeton University, NJ 08544; † Department of Biochemistry, The University of Oxford, OX1 3QU, United Kingdom; ‡ European Molecular Biology Laboratory, 69117 Heidelberg, Germany; § Institute of Genetics, University of Cologne, 50674 Germany; ** Biocenter, Institute of Developmental Biology, University of Cologne, 50674 Cologne, Germany

**Keywords:** mRNA localization, local translation, dendritic arborization neuron, multidendritic neuron, *Drosophila* peripheral nervous system

## Abstract

Localizing messenger RNAs at specific subcellular sites is a conserved mechanism for targeting the synthesis of cytoplasmic proteins to distinct subcellular domains, thereby generating the asymmetric protein distributions necessary for cellular and developmental polarity. However, the full range of transcripts that are asymmetrically distributed in specialized cell types, and the significance of their localization, especially in the nervous system, are not known. We used the *EP-MS2* method, which combines *EP* transposon insertion with the *MS2*/MCP *in vivo* fluorescent labeling system, to screen for novel localized transcripts in polarized cells, focusing on the highly branched *Drosophila* class IV dendritic arborization neurons. Of a total of 541 lines screened, we identified 55 *EP-MS2* insertions producing transcripts that were enriched in neuronal processes, particularly in dendrites. The 47 genes identified by these insertions encode molecularly diverse proteins, and are enriched for genes that function in neuronal development and physiology. RNAi-mediated knockdown confirmed roles for many of the candidate genes in dendrite morphogenesis. We propose that the transport of mRNAs encoded by these genes into the dendrites allows their expression to be regulated on a local scale during the dynamic developmental processes of dendrite outgrowth, branching, and/or remodeling.

Neurons are highly polarized cells whose dendrites and axons constitute morphologically and functionally distinct subcellular domains. The development and maintenance of these domains, which often extend long distances from the cell body, require finely tuned spatial and temporal control of gene expression. Among other strategies, neurons employ mRNA localization and on-site, on-demand mRNA translation for spatio-temporal control over gene expression autonomously from the cell body. Transcriptome studies of isolated dendrites, axons, and growth cones from cultured vertebrate neurons or neuropil have uncovered thousands of RNAs in neuronal processes, and directed analysis has also identified microRNAs that are differentially distributed between soma and dendrites or axons (reviewed in Holt and Schuman 2013). Furthermore, mRNAs appear to be abundant in neuronal processes throughout the neuronal lifespan, suggesting that local protein synthesis is used both during development and in mature, functioning neurons (Zivraj *et al.* 2010).

Misregulation of mRNA localization and local translation in neurons has been shown to contribute to multiple neurodevelopmental syndromes, including fragile X syndrome, Down syndrome, and Rett syndrome (Liu-Yesucevitz *et al.* 2011; Troca-Marin *et al.* 2012; Richter *et al.* 2015). Interestingly, dendrite dysgenesis is a common feature of these disorders, hinting that defects in local protein synthesis may impact dendrite morphogenesis. Indeed, we have previously uncovered requirements for mRNA localization, and for mRNA regulatory proteins, in dendrite patterning of *Drosophila* sensory neurons (Brechbiel and Gavis 2008; Xu *et al.* 2013; Olesnicky *et al.* 2014).

Despite the prevalence of mRNAs in neuronal processes, relatively few of these localized mRNAs have been studied in detail. As a result, our understanding of the molecular mechanisms that govern mRNA targeting to dendrites and axons and the functional implications of localization in neurons is still nascent. A long-standing obstacle to this goal has been the difficulty in visualizing localized mRNAs in the fine processes of neurons *in vivo* outside of dissociated culture systems. Detection by *in situ* hybridization has been hampered by the challenge of discriminating low endogenous neuronal transcript levels from expression in surrounding tissues. To overcome this difficulty, several studies have utilized the *MS2*/MCP system for the visualization of mRNA distributions in *Drosophila* class IV dendritic arborization (da) neurons—a subset of morphologically complex larval sensory neurons (Brechbiel and Gavis 2008; Xu *et al.* 2013). This system requires that targeted genes of interest be tagged with sequences encoding *MS2* RNA stem-loops. Concurrent expression of *MS2*-tagged transcripts and fluorescent *MS2* Coat Protein (MCP), a bacteriophage-derived protein that binds the *MS2* stem loops, results in the formation of RNP particles detectable by fluorescence microscopy (Bertrand *et al.* 1998).

Here, we adapted a previously described methodology combining the *MS2*/MCP system with EP element transposition and GAL4/UAS-driven transgene expression to characterize novel localized transcripts in the processes of *Drosophila* class IV da neurons (JayaNandanan *et al.* 2011). This method allows the unbiased identification of candidate transcripts with the capacity to localize to neuronal processes. In addition, it enables the simultaneous *in vivo* visualization of these candidates to characterize their subcellular distributions. We have identified 55 candidate transcripts capable of localizing to neuronal processes. Quantitative mRNA analysis showed that the screen detected transcripts with a wide range of expression levels. Furthermore, many of these transcripts exhibit biased localization profiles, accumulating specifically in dendrites rather than axons. *Post hoc* genomic mapping revealed that 42 of the 55 transcripts are very likely to include a portion, or all, of a known, previously annotated RNA. Subsequent gene ontology (GO) analysis suggests that, although the corresponding genes encode molecularly diverse proteins, this candidate subset is significantly enriched for genes that function in neuronal development.

A secondary RNAi screen confirmed that the expression of many candidate genes is relevant to dendrite morphogenesis. RNAi-mediated knockdown of 18 candidate genes resulted in varied defects in dendritic arborization, which we have classified as “overbranching”, “reduced branching”, and “altered branch distribution”. We suggest that the transport of mRNAs encoded by these candidates into the dendrites may be an important method of regulating gene expression on a local scale during the dynamic developmental processes of dendrite outgrowth, branching, and/or remodeling.

## Materials and Methods

### Fly strains and genetics

*EP-MS2* insertion lines were generated as described in JayaNandanan *et al.* (2011). *EP-MS2* lines were crossed to *GAL4^477^*, *UAS-mCD8:GFP/CyO*, *actin-GFP*; *UAS-MCP-RFP/TM6B* (Brechbiel and Gavis 2008; Xu *et al.* 2013) at 25°. The RNAi screen was conducted by crossing *UAS-RNAi* lines listed in Supplemental Material, Table S1 to *ppk-GAL4*, *UAS-CD4:gfp* (Han *et al.* 2011) at 29°. *Drosophila* strains are available upon request.

### EP-*MS2* screen for localized transcripts

Screening for localized transcripts was performed using a semi-intact larval preparation. An individual wandering third instar larva was immersed in a droplet of 90% glycerol on a glass slide. A small incision was made near the posterior end to extrude the gut and associated tissue. A coverslip was then pressed over the larva and the sample was imaged immediately using a Leica SPE confocal microscope with a 63×/1.4 NA oil objective. ddaC neurons from abdominal segments 3 and 4 were imaged in extended z-stacks with a 500-nm step size.

**Figure 1:**
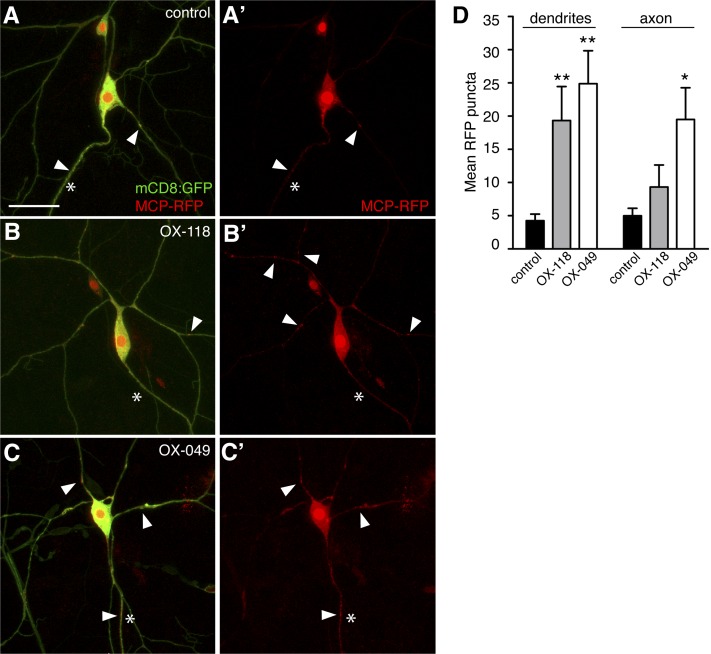
*MS2*-tagged mRNAs localize to dendrites, axons, or both. (A–C) Confocal z-series projections of class IV da neurons expressing mCD8-GFP (green), MCP-RFP (red), and *MS2*-tagged transcripts under the control of *GAL4^477^*. The green channel in the merged images was adjusted in Adobe Photoshop so that the neuronal processes are just visible. (A) Control neuron expressing mCD8-GFP and MCP-RFP but no *MS2*-tagged RNA (MCP-RFP-only). (B, C) Representative neurons from the *EP-MS2* lines OX-118 (B) and OX-049 (C). (A’–C’) Red channel corresponding to (A–C) used for particle quantification. *MS*-tagged transcripts from OX-118 preferentially localize to dendrites (B, B’), whereas transcripts from OX-049 show little preference (C, C’). Arrows indicate MCP-RFP particles; asterisks indicate axons. (D) Quantification of the average number of RNPs in dendrites or axons of at least six neurons from three to four larvae for each line shown in A–C. Error bars indicate SEM; *P ≤ 0.05, ** *P* ≤ 0.01. Scale bar = 20 µm.

At least six neurons from three or more larvae were imaged and analyzed for each *EP-MS2* line. Because particles are largely detected within the proximal dendrites and axon, nearly all particles could be captured by positioning the cell body near the center of the 174.6 × 174.6 µm image field. RNA particles were quantified from maximum z-series projections using fixed parameters in NIH ImageJ v.1.48. The red channel (for detection of MCP-RFP fluorescence) was thresholded to a fixed minimum value. The built-in particle detection function in ImageJ was then utilized to distinguish RFP-positive particles from background fluorescence levels. Particle identification parameters were set as follows: particle size, 0–20 pixels; particle circularity, 0.00–1.00. Distinct particles located within the neuronal processes (as demarcated by membrane-bound mCD8:GFP) were manually counted. Statistical significance was determined by Student’s *t*-test. Note that images shown in Figure 1 were cropped and adjusted identically in Adobe Photoshop.

### Mapping of insertions

Genomic DNA was isolated from third-instar larvae according to Huang *et al.* (2009). TAIL-PCR was performed according to the method of Liu *et al.* (1995), using three consecutive rounds of PCR with a set of degenerative primers, and a set of primers complementary to sequences within the *EP-MS2* element (Table S2). In each successive round of PCR, the *EP-MS2* primer used is downstream of the primer used in the previous round. The PCR product of the third round was sequenced and mapped to the *D. melanogaster* genome using BLAST (NCBI).

### Quantitative RT-PCR analysis of EP-*MS2* RNA expression

For each *EP-MS2* line analyzed, total RNA was purified from three third-instar larvae using the illustra RNAspin Mini kit (GE Healthcare Life Sciences) and stored at –80°. cDNA was synthesized from 100 ng of each RNA sample with RevertAid reverse transcriptase (ThermoFisher). Control reactions without reverse transcriptase were performed in parallel. Real-time PCR (qPCR) was carried out using a Bio-Rad CFX96. Each *EP-MS2* line was analyzed in triplicate reactions for each of two primer sets: primers complementary to the *EP-MS2* element, and primers complementary to *rp49*—a ribosomal protein gene used as a reference to normalize for differences in initial cDNA concentrations (Table S2). Three independent qPCR experiments were performed in all cases.

To quantify relative expression levels, ∆Ct was calculated for each *EP-MS2* line by subtracting the mean Ct-value obtained for reactions with *EP-MS2* primers from the mean Ct-value of the corresponding *rp49* reactions. Because there is no independent *EP-MS2* control sample for this experiment, we compared expression of each line to that of the mean of all 24 lines. Statistical analysis was carried out using Prism 5 software (Graphpad); statistical significance was determined using Student’s *t*-test.

### RNAi screen for dendrite morphology defects

Twelve ddaC neurons from six larvae were analyzed for each *UAS-RNAi* line (Table S1). To visualize dendrite morphology, wandering third instar larvae were mounted on slides in a 1:5 mixture of chloroform:halocarbon oil (2:1 ratio of halocarbon 95 and 200). Two 22 mm × 22 mm coverslips were placed, one on either side of the larva and a 22 mm × 60 mm coverslip was gently pressed on top. The ddaC neurons of abdominal segments 3 and 4 were imaged in z-stacks with 1 μm steps, using a Leica SPE confocal microscope with a 20× air objective. The number of terminal branches and total branch length were quantified from z-series maximum projections. Neuronal tracings used to determine branch length were made using NeuronJ (Meijering *et al.* 2004). Statistical significance was determined using Student’s *t*-test.

### Data availability

The authors state that all data necessary for confirming the conclusions presented in the article are represented fully within the article.

## Results

### A screen for localized mRNAs in Drosophila larval sensory neurons

To identify novel localized transcripts in the processes of *Drosophila* larval class IV da neurons, we took advantage of the *EP-MS2* method to generate *MS2*-tagged transcripts throughout the genome. In this method, a modified *EP* transposon containing GAL4 responsive UAS sequences and a minimal promoter followed by six *MS2* stem-loops is integrated into the genome by *P* element-mediated transposition. Because *P* elements tend to insert near the 5′-ends of genes, activation by a GAL4 driver should frequently lead to transcription of a “trapped” gene, generating an extended 5′−UTR containing the *MS2* tag. *MS2*-tagged transcripts can be visualized by simultaneous expression of MCP-RFP.

We generated a collection of *EP-MS2* insertion lines that were then crossed to the cell type-specific driver *GAL4^477^* (Grueber *et al.* 2003) for expression in class IV da neurons. Neuronal coexpression of MCP-RFP generated fluorescently labeled mRNA that was visualized in semi-intact larval preparations by confocal microscopy. Consistent with previous results, *MS2*-tagged transcripts labeled with MCP-RFP formed bright particles that could be detected in the soma and/or processes of the neurons (Figure 1). Because MCP-RFP can form nonspecific particles in da neurons in the absence of *MS2*-tagged RNA, probably due to self-aggregation (Brechbiel and Gavis 2008; Xu *et al.* 2013), we compared the number and distribution of particles observed with *EP-MS2* expression to those of control neurons expressing MCP-RFP alone (Figure 1, A and A’; “MCP-RFP-only” neurons). Particles were identified and quantified in micrographs using thresholding and particle-resolving functions in NIH ImageJ (see *Materials and Methods*; Figure 1, A–C and A’–C’).

Of 541 independent *EP-MS2* lines screened, 10% (55 lines) yielded transcripts that were significantly enriched in the processes of class IV da neurons (*P* < 0.05 when compared to MCP-RFP-only neurons; Table 1). Qualitative visual analysis of RNA particles for these lines suggested that some transcripts preferentially localize to dendrites *vs.* axons. To quantify the likelihood of polarized localization among the positive lines, we compared the number of particles detected within each neuronal compartment to the corresponding number of particles in MCP-RFP-only neurons. Twenty-seven (49%) of the positives showed significant accumulation in dendrites but not the axon (Figure 1, B–B’). These particles were detected primarily in larger proximal processes rather than in thinner distal processes. The remaining positive lines showed patterns of accumulation that also included the axon (Figure 1, C–C’).

**Table 1:** Positive candidates from *EP-MS2* screen

Nearest Gene	FlyBase ID	Line ID	Position Relative to Gene	Neurons Analyzed (*n*)	# Particles (Mean ± SEM)*^a^*	*P* Value (t-Test)*^b^*	Dendrite Enrichment*^c^*	RNAi Phenotype*^d^*
*antennal protein 10* (*a10*)	FBgn0011293	EM-802	Coding	6	23 ± 4	**	–	–
*apontic* (*apt*)	FBgn0015903	EM-842	Coding	6	21 ± 3	**	–	–
*bruno-3* (*bru-3*)	FBgn0264001	EM-402	Intron	6	19 ± 5	*	–	–
*Calnexin 99A* (*Cnx99A)*	FBgn0015622	EM-573	Coding	7	19 ± 3	*	+	–
*Calreticulin* (*Calr*)	FBgn0005585	EM-447	52 bp	6	22 ± 5	*	+	Not tested
*CG5122*	FBgn0032471	OX-061	Coding	7	44 ± 9	**	–	Not tested
*CG5261*	FBgn0031912	OX-015	60 bp	6	20 ± 4	*	+	–
*CG8177*	FBgn0036043	CO-044	1000 bp	6	33 ± 6	***	–	Decreased branching
*CG8420*	FBgn0037664	OX-064	200 bp	6	36 ± 8	**	–	–
*CG9922*	FBgn0038196	OX-012	22,000 bp	8	37 ± 8	**	–	Increased branching
*CG12535*	FBgn0029657	EM-781	Coding	6	31 ± 6	*	–	–
*CG14805*	FBgn0023514	CO-042	Intron	6	20 ± 5	*	+	–
*CG42524*	FBgn0260429	OX-118	400 bp	6	29 ± 6	**	+	Not available
*CG42855*	FBgn0262102	EM-532	Intron	9	23 ± 5	*	–	Not available
*CG43392*	FBgn0263249	OX-116	Intron	6	44 ± 9	***	–	Not available
*Chemosensory protein B 38c* (*CheB38C*)	FBgn0032888	OX-097	4700 bp	6	34 ± 9	*	–	–
*CHKov1*	FBgn0045761	CO-016	Coding	6	36 ± 8	**	–	Abnormal pattern
*coracle* (*cora*)	FBgn0010434	OX-080	5′-UTR	6	22 ± 5	*	–	Increased branching
*CR45669*	FBgn0267229	OX-063	10 bp	6	35 ± 8	**	–	Not available
*escargot* (*esg*)	FBgn0001981	OX-031	300 bp	4	33 ± 7	**	–	–
		OX-053	100 bp	6	24 ± 7	*	+*^e^*	Decreased
		OX-126	200 bp	6	26 ± 2	***		branching
*fatty acid binding protein* (*fabp*), *scheggia* (*sea*)	FBgn0037913, FBgn0037912	OX-049	2500 bp	8	44 ± 7	***	–	Increased branching
*foraging* (*for*)	FBgn0000721	EM-066	3000 bp	6	25 ± 3	***	+	Decreased branching
*frayed* (*fray*), *CG7694*	FBgn0023083,FBgn0038627	CO-033	Intron	8	29 ± 5	**	–	Increased branching; –
		OX-103	2000 bp	6	30 ± 7	**	+	
*frizzled 2* (*fz2*)	FBgn0016797	EM-019	Coding	6	18 ± 3	*	+	–
*High mobility group protein D* (*HmgD*)	FBgn0004362	CO-011	Intron	7	36 ± 7	**	–	Increased branching
*Hormone receptor-like in 39* (*Hr39*)	FBgn0261239	CO-060	2000 bp	6	18 ± 4	*	+	–
*IGF-II mRNA binding protein* (*imp*)	FBgn0262735	EM-574	1000 bp	8	20 ± 4	*	+	–
*Inositol 1,4,5-triphosphate kinase 1* (*IP3K1*)	FBgn0032147	EM-042	Intron	6	21 ± 3	**	+	Decreased branching
*Ionotropic receptor 68a* (*Ir68a*)	FBgn0036150	OX-078	4000 bp	6	20 ± 4	*	–	Increased branching
*jing interacting gene regulatory 1* (*jigr1*)	FBgn0039350	EM-562	Intron	6	27 ± 6	**	+	–
		OX-050	100 bp	6	26 ± 2	***	–*^e^*	–
		OX-052	80 bp		33 ± 6			
*Meltrin*	FBgn0265140	CO-051	Intron	6	35 ± 12	*	+	–
*Mi-2*	FBgn0262519	EM-024	Coding	6	33 ± 12	*	+	Decreased branching
*mini spindles* (*msps*)	FBgn0027948	EM-404	80 bp	4	23 ± 7	*	+	Decreased branching
*mir-315 stem loop* (*mir-315*)	FBgn0262461	EM-704	700 bp	6	21 ± 3	*	+	Not available
*Multidrug resistance protein 4 ortholog* (*Mrp4*)	FBgn0263316	EM-043	500 bp	6	19 ± 3	*	+	Not tested
*Phosphoinositide-dependent kinase 1* (*Pdk1*)	FBgn0020386	PU-007	2400 bp	6	27 ± 6	**	+	Decreased branching
*schnurri* (*shn*)	FBgn0003396	EM-503	4000 bp	6	22 ± 4	**	–	Increased branching
*shibire* (*shi*)	FBgn0003392	EM-550	Coding	6	19 ± 5	*	–	Not tested
*spitz* (*spi*)	FBgn0005672	PU-003	3300 bp	6	22 ± 3	**	+	–
*Star* (*S*)	FBgn0003310	OX-043	3500 bp	6	20 ± 3	**	–	Increased branching
*taranis* (*tara*)	FBgn0040071	OX-032	Intron	6	22 ± 4	**	+	–
*Thiolase*	FBgn0025352	CO-029	Intron	6	36 ± 9	**	–*^e^*	–
		CO-072		6	33 ± 2	***		
*three rows* (*thr*)	FBgn0003701	CO-074	Intron	6	24 ± 6	*	+	Decreased branching
*u-shaped* (*ush*)	FBgn0003963	EM-629	Coding	6	25 ± 4	**	–*^e^*	Decreased branching
		OX-047		8	20 ± 4	*		
		PU-063	Intron	6	20 ± 4	*	+	
*unable to map* (1)		EM-030		6	15 ± 2	*	+	NA
*unable to map* (2)		EM-607		6	24 ± 4	**	+	NA
*Vacuolar H^+^ ATPase 16kD subunit* (*Vha16-1*)	FBgn0262736	EM-637	Coding	6	24 ± 5	*	+	–

*EP-MS2* insertion lines are identified according to their origin (EM, EMBL, Heidelberg; OX, Oxford University; PU, Princeton University; CO, University of Cologne). For each line, the gene disrupted by the insertion (and position within the gene) or the most proximal downstream gene (and the distance from the insertion to the transcription start site) are listed. In the case of CO-033 and OX-103, *CG7694* and *frayed* share the same 5′-end and introns so it is not possible to distinguish which gives rise to the localized transcript. Only RNAi targeting the *frayed* transcript produced a phenotype. The two genes proximal to the OX-049 insertion share the same 5′-end, so both are likely to be tagged. The *UAS-RNAi* transgenes target both transcripts so it is not possible to distinguish which one is required in da neurons.

a Average number of neuronal particles for the *n* neurons analyzed.

b Comparison of mean # neuronal particles for an *EP-MS2* line to the MCP-RFP-only control (mean = 9 ± 1); * *P* < 0.05, ** *P* < 0.01, *** *P* < 0.001.

c Selective particle localization to dendrites.

d Abnormal pattern indicates altered distribution of branches, including a field coverage defect, but without statistically significant changes in overall dendrite length and number of termini.

e Data pooled from multiple lines.

### Identification of genes targeted by EP-*MS2* insertions

Genomic mapping of the positive *EP-MS2* lines by thermal asymmetric interlaced (TAIL) PCR revealed that 27 (49%) of the lines had insertions within the transcription unit of a previously annotated gene (Table 1). An additional 15 lines had insertions ≤ 1 kb upstream of a transcriptional start site, likely generating a transcript with an extended 5′UTR. These sets included multiple independent insertions for several genes—*escargot*, *jing interacting gene regulatory 1*, and *u-shaped*—suggesting that these loci may be insertional hot-spots. Indeed, *escargot* was previously identified as a hot-spot locus (Spradling *et al.* 1999). For 11 lines, the nearest downstream transcription unit was ≥ 1 kb away, although whether the *MS2*-tagged transcripts include sequences from these genes or derive only from the intergenic region is not clear. In the latter case, we presume that these intergenic regions contain sequences that, if transcribed, can function as cryptic localization elements. Finally, we were unable to map two insertions. In total, 47 different genes were identified; in two cases two overlapping genes were targeted by the same *EP-MS2* insertion.

We also mapped 23 negative lines to determine whether they represented insertions within genes or primarily intergenic insertions. Sixteen (69%) had insertions within or ≤ 1 kb upstream of an annotated transcription unit (Table 2). Thus, the majority of negative lines likely represent productive insertions that could generate *MS2*-tagged transcripts but that did not show specific localization patterns of the type we describe above. This analysis also revealed three cases in which the same genes were identified among both positive and negative lines. For *Multidrug resistance protein 4 ortholog* and *escargot*, both positive and negative lines contained insertions near the transcription start site and the negative lines produced transcripts (see below), suggesting that those insertions may lack *MS2* stem-loops or be otherwise defective. The overlapping genes *fatty acid binding protein* (*fabp*) and *scheggia* (*sea*) were identified by insertions residing 2.4 kb (positive) and 2.1 kb (negative) upstream. The finding that neuronal knockdown of *fapb*/*sea* affects dendrite arbor morphology (see below; Table 1) suggests that the negative line similarly contains a nonproductive insertion, although we cannot rule out the possibility that the more upstream insertion is a false positive.

**Table 2:** Negative lines mapped and/or analyzed for expression levels

Nearest Gene	FlyBase ID	Line ID	Position Relative to Gene (bp)	qRT-PCR
*bruchpilot* (*brp*)	FBgn0259246	EM-405	Intron	
*CG1358*	FBgn0033196	OX-112	Coding	
*CG3927*	FBgn0034739	OX-017	24,000	
*CG5151*	FBgn00366576	EM-836	100	
*CG5381*	FBgn0032218	PU-017	Intron	
*CG8419*	FBgn0031999	EM-786	1100	√
*CG8420 CR45196*	FBgn0264439	OX-014	1100	
*CG9384*	FBgn0036446	EM-648	2500	
*CG15358*	FBgn0031373	PU-028	1900	
*CG42855*	FBgn0262102	EM-951	100	
*chameau* (*chm*)	FBgn0028387	OX-107	Intron	√
*circadian trip* (*ctrip*)	FBgn0260794	EM-758	Intron	√
*CR43174*	FBgn0267794	OX-087	300	
*escargot (esg)*	FBgn0001981	EM-628	100	√
*fatty acid binding protein* (*fabp*), *sheggia* (*sea*)	FBgn0037913, FBgn0037912	CO 066	2100	
*found in neurons* (*fne*)	FBgn0086675	EM-690	Intron	
*glycerol-3-phosphate dehydrogenase* (*Gpdh*)	FBgn0001128	PU-013	5′-UTR	
*Lk6*	FBgn0017581	EM-777	Coding	√
*longitudinal lackings* (*lola*)	FBgn0005630	CO 014	Intron	
*Mi-2/SU*(*TpI*)	FBgn0262519	EM-544	Intron	
*Multidrug resistance protein 4 ortholog* (*Mrp4*)	FBgn0263316	EM-733	150	√
*RhoGEF64C*	FBgn0035574	OX-048	5′-UTR	
*SIFamide receptor* (*SIFaR*)	FBgn0038880	EM-742	Intron	
		EM-566		√
		EM-913		√
		PU-031		√
		PU-032		√
		PU-048		√
		PU-076B		√

a qRT-PCR results are shown in Figure 2.

### EP-*MS2* expression levels do not correlate with RNA localization

The difference between positive and negative lines could reflect differences in transcript level and thus ease of detection rather than true differences in localization. To determine whether positive lines are generally associated with high transcript levels, we compared RNA expression levels among a set of 12 positive and 12 negative *EP-MS2* lines using quantitative RT-PCR (qRT-PCR). Expression of *MS2*-tagged transcripts was activated in larval class IV da neurons using *GAL4^477^*, and qRT-PCR was performed on RNA isolated from whole larvae with primers specific for expressed regions of the *EP-MS2* transgene. To facilitate comparison among the different lines, expression levels were displayed relative to the average expression level of the 24 lines (Figure 2). Statistical analysis showed that there is no significant difference in the mean expression level of lines determined to be positive *vs.* lines determined to be negative (*P* = 0.61; Figure 2). We therefore conclude that there is no correlation between the level of expression and the categorization of a transcript as localized, and that the screen has the ability to detect localized transcripts whether highly expressed, or expressed at a low level.

**Figure 2:**
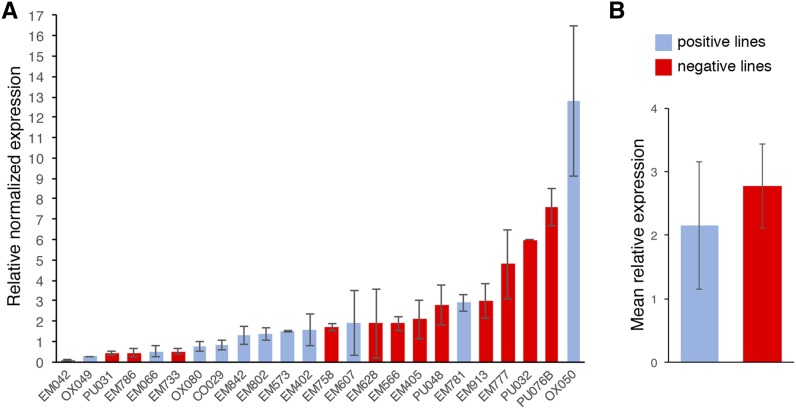
qRT-PCR analysis of *EP-MS2* line RNA expression. (A) Quantification of the relative expression levels of the indicated *EP-MS2* lines by real-time PCR of cDNA prepared from each line. The value shown represents the mean value for the indicated line relative to the mean of all 24 lines analyzed (see *Materials and Methods*). (B) Comparison of the mean expression levels of the positive and negative lines analyzed in (A). Error bars indicate standard error of the mean (SEM).

### Enrichment of developmental genes among the positive candidate group

Functional sorting of the genes tagged in positive lines using the Database for Annotation, Visualization and Integrated Discovery (DAVID v. 6.7; Huang da *et al.* 2009a, 2009b) revealed enrichment for genes encoding proteins involved in neuronal development and physiology as well as several other developmental processes (Table 3). The category of nervous system development includes a diverse group of genes encoding transcription factors and DNA binding proteins (*escargot*, *High mobility group protein D*, *apontic*, *schnurri*, and *Mi-2*), a serine-threonine kinase (*frayed*), an apico-basal polarity protein (*coracle*), RNA binding proteins (*apontic* and *IGF II mRNA binding protein*), a transferase (*Star*), a nucleotide exchange factor (*RhoGEF64C*), and membrane receptors (*spitz* and *frizzled2*). In sum, localized transcripts trapped by EP-*MS2* transposons encoded functionally diverse developmental proteins.

**Table 3:** Functional annotation of candidate genes

GO Term	Number of Genes	Fold Enrichment	Genes
Peripheral nervous system development (GO:0007422)	7	19.1	*Calr*, *S*, *esg*, *fray*, *shn*, *spi*, *thr*
Neuron development (GO:0048666)	6	4.4	*Mi-2*, *HmgD*, *S*, *fray*, *fz2*, *spi*
Neuron differentiation (GO:0030182)			
Neurological system process (GO:0050877)	8	3.5	*Calr*, *S*, *a10*, *apt*, *for*, *fray*, *shn*, *shi*
Regionalization (GO:0003002)	7	3.7	*CG14709*, *cora*, *msps*, *shn*, *spi*, *tara*, *ush*
Enzyme linked receptor protein signaling pathway (GO:0007167)	5	8.3	*Pdk1*, *S*, *shn*, *spi*, *ush*
Open tracheal system development (GO:0007424)	7	11.0	*S*, *apt*, *cora*, *esg*, *shi*, *spi*, *thr*
Respiratory system development (GO:0060541)			
Epithelial development (GO:0060429)	7	8.2	*apt*, *cora*, *esg*, *fray*, *shn*, *shi*, *thr*, *ush*

Analysis of genes in Table 1 was performed using DAVID. The most highly represented functional annotation categories are listed.

### Secondary screen of gene function in neuronal morphogenesis

To determine whether genes identified in the *EP-MS2* screen function in class IV da neuron development, we knocked down expression of the majority (38) of these genes in the neurons by transgenic RNAi. *UAS-RNAi* was expressed using the class IV da neuron-specific *ppk-GAL4* driver, and coexpression of the CD4:GFP membrane marker (Han *et al.* 2011) allowed visualization of neuronal morphology. Two independent *UAS-RNAi* lines were tested for each gene in order to minimize false positive and negative results. In all cases, both lines produced similar phenotypes.

**Figure 3:**
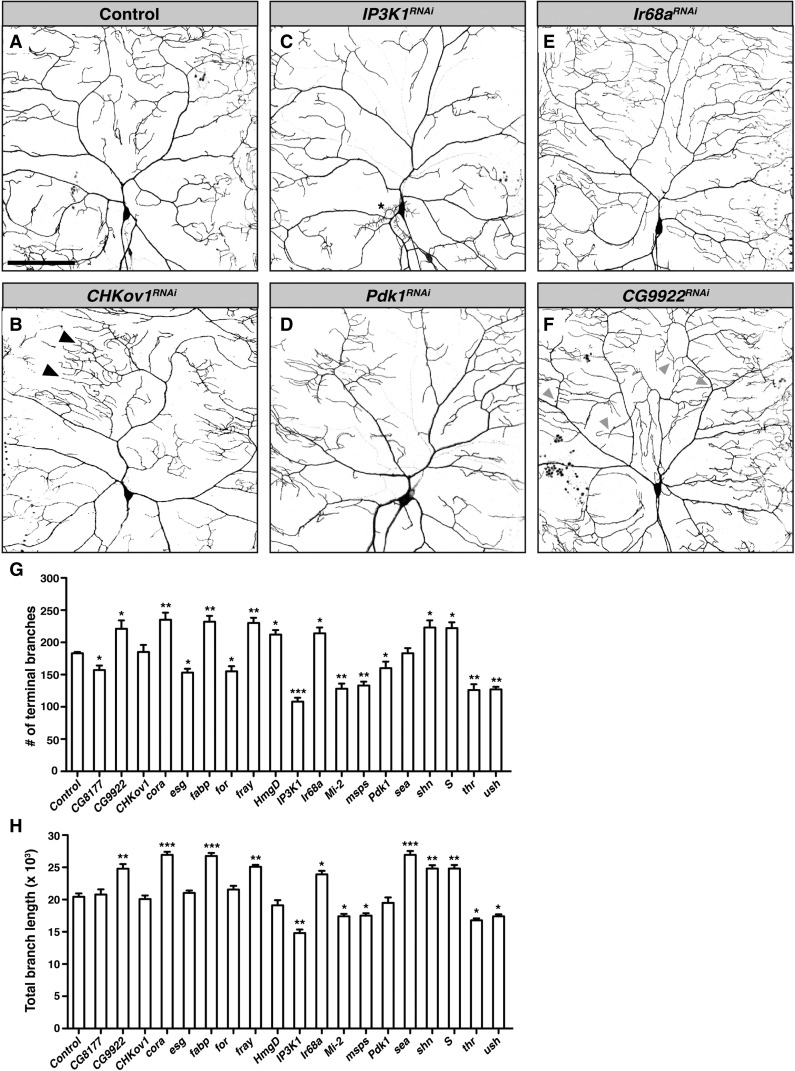
RNAi screen for dendritic arborization defects. (A–F) Confocal z-series projections of representative ddaC neurons with *ppk-GAL4* driving expression of *UAS-CD4-gfp* alone (control neuron; A) or together with the indicated *UAS-RNAi* transgene. (B) Representative neuron exhibiting abnormal patterning with patchy overbranching (arrowheads) not reflected by the quantitative measures used. (C, D) Representative images of neurons exhibiting underbranched phenotypes. Class III da neurons are occasionally labeled by *ppk-GAL4* (asterisk). (E, F) Representative images of neurons with overbranched phenotypes. Arrowheads indicate dendrite crossing events, signifying a failure of self-avoidance. (G–H) Quantification of branch length and number of terminal branches in neurons of each genotype. Two neurons from each of six larvae (12 neurons in total) were analyzed for each RNAi line. Two neurons from each of 10 larvae (20 in total) were analyzed for the control. Values shown are mean ± SEM; * *P* ≤ 0.05, ** *P* ≤ 0.01, *** *P* ≤ 0.001. Scale bar = 100 μm.

Two parameters that reflect dendritic arbor branching, total dendrite length and the number of dendritic terminal branches, were quantified from confocal z-series projections of RNAi-expressing neurons. In total, knockdown of 18 genes (47% of those tested) produced a dendrite morphogenesis phenotype. A decrease in branching relative to control neurons was most frequently observed (nine genes; Table 1 and Figure 3, G and H). For example, knockdown of two kinases, *Pdk1* and *IP3K1*, resulted in loss of higher order branches, and consequent reduction in overall coverage of the receptive field (Figure 3, C and D). Interestingly, Pdk1 protein has previously been shown to localize to the *Drosophila* neuromuscular junction, where it positively regulates synaptic bouton size (Cheng *et al.* 2011). Our data suggest that Pdk1 may also act locally in da neuron dendrites. In contrast, knockdown of seven genes resulted in increased branching relative to control neurons (Table 1 and Figure 3, G and H). Examples of genes displaying this phenotype include the ionotropic receptor-encoding gene *Ir68a* and the poorly characterized gene *CG9922* (Figure 3, E and F). Notably, knockdown of *CG9922* also caused defects in dendritic self-avoidance (Figure 3F), suggesting a broad regulatory function in dendrite morphogenesis. In several cases, we observed alterations in branch length that were not explained by changes in branch number (Figure 3, F and G). Finally, we observed one instance of patchy defects in patterning of the arbor and spacing of branches that was not reflected by quantification of total dendritic length or terminal branch number (Figure 3, B, G, and H).

## Discussion

By adapting a previously described EP transposon-*MS2* aptamer tagging and visualization method (JayaNandanan *et al.* 2011), we have identified 47 genes that encode mRNAs with the capacity to localize to the axonal and/or dendritic processes of *Drosophila* class IV da neurons. Although confirming the endogenous localization of these candidates via *in situ* hybridization has not yet been possible due to technical limitations, the enrichment of genes with known roles in neuron development and the functional requirement for many of them in dendritic arborization demonstrated by RNAi support the validity of our initial results.

### Utility of the EP-*MS2* method

We took advantage of the tendency of MCP-labeled RNAs to form discrete RNP particles in class IV da neurons (Brechbiel and Gavis 2008; Xu *et al.* 2013) to quantitatively identify transcripts with statistically significant localization patterns. It is likely, however, that some truly localized transcripts were missed due to a high degree of variability in particle numbers among neurons from the same line. Such variability was particularly evident in some lines reimaged after months or years, suggesting that *EP-MS2* insertions at some genomic loci may be susceptible to silencing over time. Screens performed in parallel for RNAs localized in the ovary, terminal cells of the trachea and in neuromuscular junction (NMJ) encountered difficulties due in part to the lack of discrete, quantifiable particles and confounding background fluorescence from the surrounding tissue layers. The class IV da neuron screen was indeed advantaged by the ease with which these very superficial neurons could be imaged. Despite the limitations, this method proved effective at identifying a collection of genes whose function may be locally required for neuronal development and/or function.

### Localized mRNAs encode functionally diverse proteins

Consistent with transcriptome studies in neuronal processes (for examples, see Cajigas *et al.* 2012; Minis *et al.* 2014), the set of positive candidates identified in our screen comprise genes encoding proteins with diverse functions, including structural proteins, cell-surface receptors, intracellular signaling pathway components, and even transcription factors. Although the identification of transcription factors and DNA binding proteins within this candidate pool may seem surprising, mRNAs encoding several transcription factors including cAMP response element binding protein (CREB) have previously been shown to be axonally and/or dendritically localized. CREB synthesized in the processes is then transported retrogradely to the nucleus, linking events in the periphery to transcriptional responses (Eberwine *et al.* 2001; Jung *et al.* 2012). Interestingly, the chromosomal protein High mobility group protein D and the transcription factor Escargot, both identified in the screen, have previously been implicated in neuronal morphogenesis: High mobility group protein D was found to regulate branching of class I da neurons (Parrish *et al.* 2006), while Escargot has been implicated in axonal development (Ramat and Gho 2013).

Functional sorting of our positive candidates revealed that at least 10 had previously been shown to play a role in nervous system development, with many contributing directly to the development of the peripheral nervous system. Our secondary RNAi screen confirmed functions for seven of these genes in class IV da neuron dendrite morphogenesis, and further identified 12 new genes that also regulate this process. These new candidates included a polarity protein, a microtubule-associated protein, several kinases, and an additional transcription factor, hinting at the possibility that the local translation of many functionally diverse proteins may be regulated coordinately to orchestrate dendrite morphogenesis.

### mRNA localization in dendrite morphogenesis and beyond

As described above, results from the secondary RNAi screen indicate that a large number of the identified genes influence dendrite morphogenesis in class IV da neurons. While many studies have investigated requirements for the localization of mRNAs and/or RNA binding proteins and translation factors during the morphogenesis and remodeling of postsynaptic dendritic spines (Mikl *et al.* 2010; Thomas *et al.* 2014), the role of mRNA localization and local translation in the gross morphogenesis of dendritic arbors has not been addressed to the same extent. Our previous work utilized the *MS2*/MCP system to demonstrate that the localization of *nanos* mRNA is essential for proper dendritic branch morphogenesis in class IV da neurons (Brechbiel and Gavis 2008). The current results build on those initial findings to suggest that mRNA localization may be utilized widely for the regulation of dendrite growth and branching.

Genes involved in other dynamic processes such as sensory processing and adaptation may also be represented among the positive *EP-MS2* lines but would not have been detected in our RNAi screen, which focused specifically on dendrite morphology. Furthermore, for 26 lines, we observed RNA particles in axons as frequently as in dendrites; the genes tagged in these lines may play important roles in axon development or function. Because class IV da neuron axons fasciculate with each other and with other peripheral neurons, visualization of axonal morphology requires analysis of single neuron mutant clones. Future mutant studies using mosaic analysis with a repressible cell marker (MARCM) may reveal roles for these localized RNAs in axonal development and function.

## Supplementary Material

Supplemental Material
