# Supplementary material for: A Genome-Wide Screen for Dendritically Localized RNAs Identifies Genes Required for Dendrite Morphogenesis
Source: G3 (Bethesda). 2016 Jun 1;6(8):2397–405. doi: 10.1534/g3.116.030353 (PMC4978894; doi:10.1534/g3.116.030353)
Supplement: Supplemental Material [file supp_6_8_2397__index.html]

A Genome-Wide Screen for Dendritically Localized RNAs Identifies Genes Required for Dendrite Morphogenesis — Supplemental Material 

# A Genome-Wide Screen for Dendritically Localized RNAs Identifies Genes Required for Dendrite Morphogenesis

## Supplemental Material for Misra *et al.*, 2016

**Files in this Data Supplement:**

- Table S1 - *UAS-RNAi* lines tested. (.pdf, 23 KB)
- Table S2 - Primers. (.pdf, 10 KB)
