# Supplementary material for: A Genome-Wide Screen for Dendritically Localized RNAs Identifies Genes Required for Dendrite Morphogenesis
Source: G3 (Bethesda). 2016 Jun 1;6(8):2397–405. doi: 10.1534/g3.116.030353 (PMC4978894; doi:10.1534/g3.116.030353)
Supplement: Supplemental Material [file supp_g3.116.030353_TableS1.pdf]

Table S1 ***UAS-RNAi* lines tested**

Gene	RNAi Lines
antennal protein 10 (*a10*)	TRiP HMS02394
apontic (*apt*)	TRiP JF02134; VDRC 4289
bruno-3 (*bru-3*)	TRiP HMS02970, HMC03283
Calnexin 99A (*Cnx99A*)	TRiP HMS02304, HMJ22149
CG12535	VDRC 49936, 107672
CG14805	VDRC 105616, 17477
CG5261	TRiP HMS02170, GLC01404
CG7694, *frayed* (*fray*)	CG7694: VDRC 25520, 108995<br>fray: TRiP HMJ02228, GL00704
CG8177	TRiP HMC03399; VDRC 39492
CG8420	VDRC 28635, 104679
CG9922	VDRC 35465, 105294
Chemosensory protein B 38c (*CheB38C*)	TRiP HMC04233; VDRC 35465
ChKov1	VDRC 27290
coracle (*cora*)	TRiP HMS01413; VDRC 9787

escargot (*esg*)	TRiP HMS01413, HMS02538
fatty acid binding protein (*fabp*), *scheggia* (*sea*)	fabp: TRiP HMS01163<br>sea: TRiP HMS00936; VDRC 109169
foraging (for)	TRiP GL00026, JF01134
frizzled 2 (*fz2*)	TRiP JF01259, JF02722
High mobility group protein D (*HmgD*)	TRiP JF01302; VDRC 19026
Hormone receptor-like in 39 (*Hr39*)	TRiP HMS00018, JF02432
IGF-II mRNA binding protein (*Imp*)	TRiP HMS01168, HMC03794
Inositol 1,4,5-triphosphate kinase 1 (*IP3K1*)	TRiP HM04042; GL00198
Ionotropic receptor 68a (*Ir68a*)	VDRC 8957, 106708
jing interacting gene regulatory 1 (jigr1)	TRiP HMJ221503, JF02211
Meltrin	TRiP HMJ22461; VDRC 102041
Mi-2 ortholog (*Mi-2*)	TRiP HMC03329, HMS00301
mini spindles (*msps*)	TRiP HMS01906, JF01613
Phosphoinositide-dependent kinase 1 (*Pdk1*)	TRiP HMS01250, GL00489
schnurri (*shn*)	VDRC 3226, 105643
spitz (*spi*)	VDRC 3920, 103817
Star (*S*)	TRiP GL00686; VDRC 109838

taranis (*tara*)	TRiP JF01421; VDRC 107508
Thiolase	VDRC 105500, GD1226 (no longer available)
three rows (*thr*)	VDRC 38089, 48344
u-shaped (*ush*)	TRiP HMS00744, HM05193
Vacuolar H^+ *ATPase 16kD subunit* (*Vha16-1*)	TRiP HMS02171; VDRC 49290
