# Supplementary material for: A Genome-Wide Screen for Dendritically Localized RNAs Identifies Genes Required for Dendrite Morphogenesis
Source: G3 (Bethesda). 2016 Jun 1;6(8):2397–405. doi: 10.1534/g3.116.030353 (PMC4978894; doi:10.1534/g3.116.030353)
Supplement: Supplemental Material [file supp_g3.116.030353_TableS2.pdf]

Table S2

Primers	
Primers for TAIL PCR	
5' end *EP-MS2* element primer	Cycle 1 Plac1: 5'-CACCCAAGGCTCTGCTCCCACAAT-3'<br>Cycle 2 EP5-2: 5'-TACTCCAGTCACAGCTTTGCAGCA-3'<br>Cycle 3 Sp1: 5'-ACACAACCTTTCCTCTCAACAA-3'
3' end *EP-MS2* element primers	Cycle 1 Pry1 out: 5'-ATTCAAACCCCACGGACATGCTAAGG-3'<br>Cycle 2 Pry4a: 5'-ACAATCATATCGCTGTCTCACTCAG-3'<br>Cycle 3 SpEP1a: 5'-CGACACTCAGAATACTATTCCTTTCAC-3'
Random primers	AD1: 5'-NTCGA(G/C)T(A/T)T(G/C)G(A/T)CTT-3'<br>AD2: 5'-NGTCGA(G/C)(A/T)ANCANAGA-3'<br>AD3: 5'- (A/T)GTGNAG(A/T)ANCANAGA-3'
Primers for qPCR	
EP-MS2 element primers	Forward: 5'-CATGGGACGTCGACCTGAG-3'<br>Reverse: 5'-CTAGGCAATTAGGTACCTTAGGAT-3'
rp49 primers	Forward: 5'-GCTAAGCTGTCGCACAA-3'<br>Reverse: 5'-TCCGGTGGGCAGCATGTG-3'
